# Supplementary material for: Gold-Decorated Platinum and Palladium Nanoparticles as Modern Nanocomplexes to Improve the Effectiveness of Simulated Anticancer Proton Therapy
Source: Pharmaceutics. 2021 Oct 18;13(10):1726. doi: 10.3390/pharmaceutics13101726 (PMC8539939; doi:10.3390/pharmaceutics13101726)
Supplement: Supplementary file 1 [file pharmaceutics-13-01726-s001.zip › pharmaceutics-1418042-supplementary.pdf]

pharmaceutics

MDPI

Supplementary Materials: Gold-Decorated Platinum and Palladium Nanoparticles as Modern Nanocomplexes to Improve the Effectiveness of Simulated Anticancer Proton Therapy

Bartosz Klebowski, Malgorzata Stec, Joanna Depciuch, Adrianna Gałuszka, Anna Pajor-Swierzy, Jarek Baran and Magdalena Parlinska-Wojtan

The figure below shows the results of nanoparticle cytotoxicity studies against all four cell lines after different incubation times (3, 18, 24 and 42 h). It can be seen that for both types of NPs (even for higher concentrations), no significant decrease in survival was observed for the incubation of 3 h. In turn, after 18, 24 and 42 h, a decrease in cell proliferation was observed. However, the differences in survival for these three incubation times do not differ significantly. Since no decrease in cell proliferation was observed for longer incubation times, we have chosen time 18 h to study the effect of the combined therapy on colon cells.

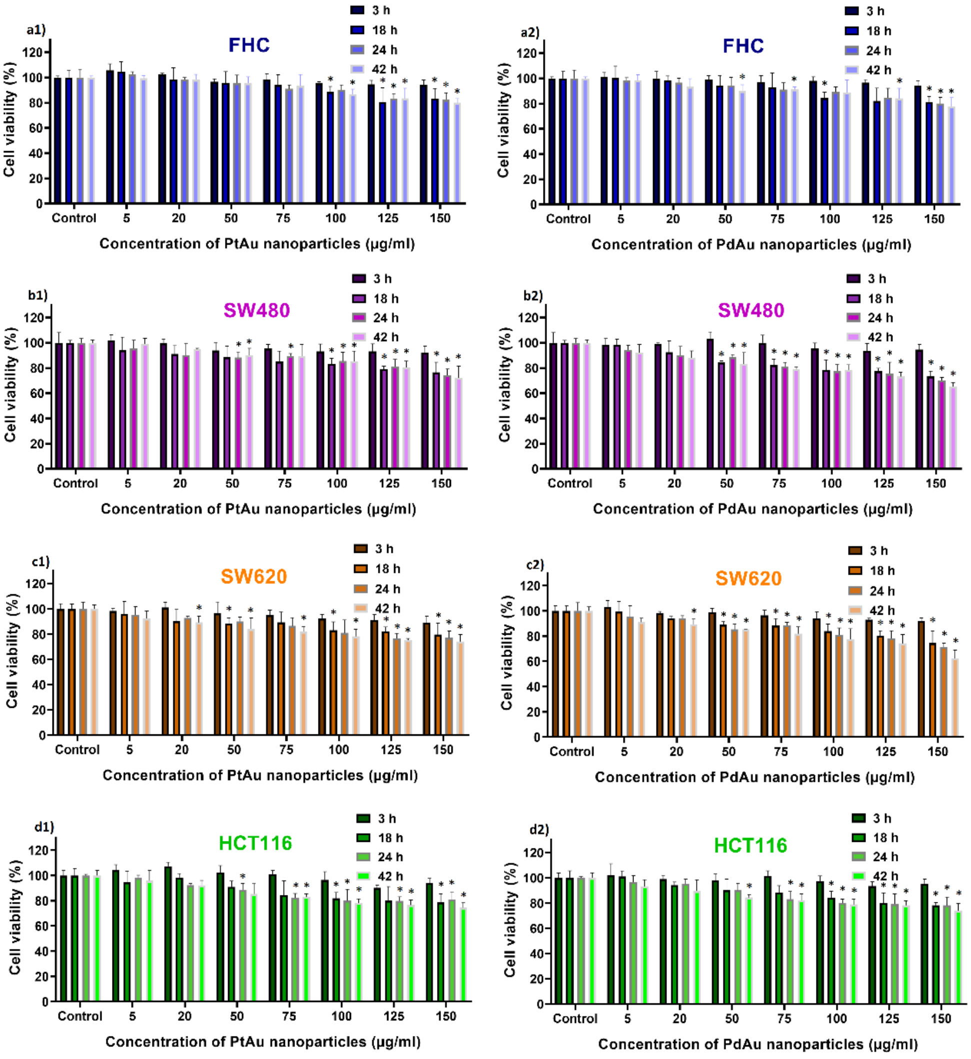

Figure S1. Cytotoxicity of PtAu NPs (**a1, b1, c1, d1**) and PdAu (**a2, b2, c2, d2**) against tested cell lines after 3, 18, 24 and 42 h of incubation. Data were considered significant when *$p < 0.05$ vs control.
